# Reducing Over-Interviewing in the Anesthesiology Residency Match

**DOI:** 10.7759/cureus.17538

**Published:** 2021-08-29

**Authors:** Ephy R Love, Franklin Dexter, Jason I Reminick, Suzanne B Karan

**Affiliations:** 1 The Bredesen Center, Data Science Engineering, University of Tennessee, Knoxville, USA; 2 Anesthesia, University of Iowa, Iowa City, USA; 3 SJ Medconnect, Thalamus, Santa Clara, USA; 4 Anesthesiology and Perioperative Medicine, University of Rochester, Rochester, USA

**Keywords:** residency recruitment, interviewing, graduate medical education, residency match, residency application process

## Abstract

Background

The U.S. residency recruitment process is expensive and time-consuming because of application inflation and over-invitation.

Objective

Using interview and match data, we quantify the predicted effects if anesthesiology residency programs excluded interviews for applicants who are very unlikely to match.

Methods

We previously published the validity and accuracy of the logistic regression model based on data from interview scheduling software used by 32 U.S. anesthesiology residency programs and 1300 applicants from 2015-18. Data used were program region, applicant address, numbers of interviews of the interviewee, medical school US News and World Report (USNWR) rank, the difference between United States Medical Licensing Exam (USMLE) Step 1 and 2 Clinical Knowledge (CK) scores, and the historical average of USMLE scores of program residents. In the current study completed in 2020, the predicted probabilities and their variances were summed among interviewees for 30 deidentified programs.

Results

For anesthesiology, the median residency program could reduce their interviews by 16.9% (97.5% confidence interval 8.5%-24.1%) supposing they would not invite applicants if the 99% upper prediction limit for the probability of matching was less than 10.0%. The corresponding median savings would be 0.80 interviews per matched spot (0.34-1.33). In doing so, the median program would sustain a risk of 5.3% (97.5% confidence interval 2.3%-7.9%) of having at least one interviewee removed from their final rank-to-match list.

Conclusion

Using novel interview data and analyses, we demonstrate that residency programs can substantively reduce interviews with less effect on rank-to-match lists. The data-driven approach to manage marginal interviews allows program leadership to better weigh costs and benefits when composing their annual list of interviewees.

## Introduction

United States residency programs have seen an increased number of applications and interviews in the last five years (denoted “application inflation”), which imposes a large cost burden to applicants and programs alike [1-2]. Video-based interviewing is reported to be an acceptable alternative to in-person interviews [3-5] that decreases costs of travel for applicants. However, application inflation requires ever-increasing faculty time to review [6]. The objective of our current study is to quantify the extent to which conducting fewer interviews is feasible [7].

Our prior work demonstrated the novel use of interview data from anesthesiology residencies to quantitate the effect of an applicant being from the same state on the probability of matching at a residency program [8]. An applicant living in the same state as the residency program could have 5.42 fewer total interviews (97.5% confidence interval 3.02-7.81) [9] while having the same odds of matching; the state was the significant predictor, not matching medical school [8]. This finding was novel and timely given that a survey performed contemporaneously of anesthesiology program directors found that among factors considered when selecting an applicant for an interview, none of the 27 listed included the applicant’s residence within the USA [10]. Furthermore, a 2020 National Residency Match Program (NRMP) program director survey did not list the geographic location as a choice when soliciting preferences in factors used to invite and/or rank applicants for all included specialties [11]. In contrast, a 2019 NRMP applicant survey revealed that geographic location is one of the major factors prioritized by applicants when ranking programs [12]. A recent survey of obstetrics and gynecology residents showed that location was the most important criteria for where residents match [13].

Expanding on our work [8], we sought to quantify the effects on residency programs of excluding interviews for applicants whom they are very unlikely to match [14]. In other words, and as previously editorialized [15], by decreasing unnecessary interviews, programs and applicants might save time and money with accompanying minimal change in match outcomes, including applicants matching and programs filling. In this paper, we quantify the extent to which this is true.

## Materials and methods

The study was reviewed by the University of Iowa Institutional Review Board (IRB 201909708). Prior to submitting this for review, interviewee and residency program data were gathered from four software platforms (Thalamus, Doximity, ACGME, and U.S. News and World Report) and de-identified before analysis (described below). The University of Iowa IRB determined that the study of this de-identified data does not meet the regulatory definition of human subjects’ research, and therefore did not require review of the IRB or written consent from interviewees or programs. Thalamus is a cloud-based, graduate medical education (GME) interview scheduling software and management platform (SJ MedConnect, Inc. dba ThalamusGME, Santa Clara, CA; https://thalamusgme.com/) [16].

The current study extends work completed in 2020 [8], wherein we performed logistic regression modeling for the probability of an interview resulting in a match. Data used were whether the interviewee was currently located in the same state as the program, regional location, numbers of interviews, medical school rank, and the difference between United States Medical Licensing Exam (USMLE) Step 1 and 2 Clinical Knowledge (CK) scores and the programs’ historic average for residents. The probability of an interview (an interviewee and program combination) resulting in a match was estimated using: a combination of the average of the USMLE Step 1 and Step 2 CK scores differenced the mean of these averages for the program, an indicator variable of whether or not the interviewee resides in the same state as the program based on interview “Current Address” on their Electronic Residency Application Service (ERAS) application, a control for the region of the program, a count of recorded interviews, and the Doximity rank of the interviewing program. To determine interview excess, the subsequently described analyses were performed, summing among interviews at each program.

Analysis

In order to assure program anonymity, two of the 32 programs in the original paper were removed because these programs each had fewer than 10 interviews. The two programs were removed after parameters and variances were estimated. The 30 programs studied in this work come from 19 unique states (40 US States and DC have at least one anesthesiology program). The median anesthesiology program has seven other programs in their state. The programs in our sample also had a median of seven other programs in their states.

Our current study quantifies the potential reduction in surplus interviews per program, and the associated risk, through the implementation of a decision rule based on an estimated probability of matching. To achieve this, we needed a method to compute the number of interviews that would cease at a given specified certainty and threshold as well as a method of computing the associated risk of a change in the final match list. We computed intervals for each prediction at multiple levels of certainty [8]. Whereas certainty about a population parameter can be given in the form of a confidence interval, prediction intervals are the analogous structure for denoting certainty about the prediction of a single value (in this case, the probability of an interviewee matching with a program). The upper limit of the prediction interval then seeks to give the highest probability of matching that could have in fact been associated with an interviewee-program-pairing.

We use *e_i_* to refer to the binary prediction from the logistic regression that interview *i* resulted in a match. The predictions and prediction intervals of the logistic regression are initially calculated on a log-odds (‘logit’) scale. That means that with *e_i_* being a prediction from the logistic regression, *e_i _= log (p_i_/1-p_i_) where p_i_ is a probability*. The inverse logit function *L^-1^(e_i_) = exp(e_i_)/exp(e_i_)+1 = p_i_* was used to recover the probabilities *p_i_*.

Our objective was to estimate the proportion of interviews conducted per program and with a low probability of a match. To decide whether an interview was surplus, choices of a width of prediction interval as well as a threshold of minimum probability were made. Prediction intervals were constructed through a choice of certainty $\delta$, an interval on the Z-distribution (standard normal), with larger values of $\delta$ corresponding to increased certainty that the true prediction lies within those limits. We examined two values $\delta$: 2.58 and 1.96, which correspond to 99% and 95% intervals, respectively. The standard $\delta$ s are scaled by multiplication with each interview’s estimated standard deviation *s_i_* in the logit scale and added to *e_i_* to form an upper limit on the probability *l_i _= e_i_ + s_i_ x $\delta$*. The inverse logits of the interval were used to attain probabilities. The upper probability limits were then compared on the prediction intervals with a chosen probability threshold *t*; i.e., we removed interviews where p<t. Thresholds of 0.01 (1%), 0.05 (5%), and 0.1 (10%) were examined.

**Figure 1:**
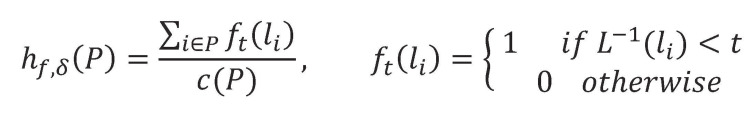
Equation 1

Since this study sought to quantify risk and reward for programs, estimates of the program among interviewees were aggregated. Here* f_t_* represented an indicator function corresponding to whether a prediction interval upper limit fell below the minimal threshold to be considered for an interview. The sum of *f_t _*over a program’s interviews yielded the total interviews to be removed for that program, at a choice of confidence level and threshold. Let *c(P)* denote the size of *P* (total interviews for a program). The number of interviews cut for each program are defined via a method for computing in Equation (1) (Figure 1), where *h_f _*sums over the interviews in *P* a set of interviews belonging to a residency program.

Including *c(P)* in the denominator of *h_f_* accounts for the variation in the number of interviews among programs. Equation (1) (Figure 1) was repeated for each of the programs.

**Figure 2:**
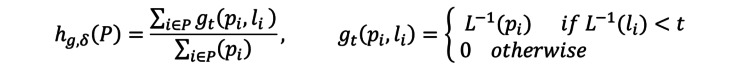
Equation 2

To estimate the risk associated with removing these interviews, the sum of prediction interval upper limits was calculated for the removed interviews, given in *g_t_*. Note, the combined probabilities of candidates not matching from the sum of probabilities of the program were not modeled nor removed. Because the probability combined is the sum of probabilities minus the probability of combinations, an upper limit on the risk (the risk is guaranteed to be less than or equal to the values reported) was computed. The computations follow the same pattern as above but sum the probabilities rather than interviews, shown in Equation (2) (Figure 2).

The denominator in *h_g_* accounts for the variation in total probabilities of matching among programs by summing over the maximum likelihood estimated probabilities of all interviews conducted by the program.

Finally, we used the numerator of Equation (1) and the denominator of Equation (2) to estimate the reduction in interviews counted per matched spot of the program.

Estimating the characteristics of the median program

The calculated quantities from the logistic regression model for each of the 30 programs are provided in the data workbook provided in the supplemental content. We calculated the reduction in interviews and associated cumulative risk of implementing a cutoff for each program. We chose a useful set of cutoffs to include in the workbook and summarize these findings in Table 1, which reports the median (50th percentile program) of *h_t_ and g_t_*. The table shows 97.5% two-sided non-parametric conservative confidence intervals for the median by reporting the eighth and twenty-first of the ranked values of *h_t_* (sum of interviews) and *g_t_* (sum of probabilities) [17]. Non-parametric confidence intervals were used because of the skewness of the estimated risks among programs, as shown in the supplemental workbook. We used 97.5% confidence intervals because the two endpoints in each row of Table 1 are directly associated. We similarly used a 97.5% confidence interval for the interview reduction per matched spot because it is a separate way of reporting the other two ratios.

**Table 1:** Parameters used for decision on exclusion of marginal interviews

Variable	Prob	Position	Median	Lower limit	Upper limit	Program, Sum Probabilities	Prob	Position	Median	Lower limit	Upper limit	Applicants, Sum Interviews
u99Sum10	u99ProbSum10	22	5.3%	2.3%	7.9%	5.3% (2.3%, 7.9%)	u99IndSum10	25	16.9%	8.5%	24.1%	16.9% (8.5%, 24.1%)
u99Sum05	u99ProbSum05	23	0.8%	0.3%	1.6%	0.8% (0.3%, 1.6%)	u99IndSum05	26	4.9%	1.5%	8.9%	4.9% (1.5%, 8.9%)
u99Sum01	u99ProbSum01	24	0.0%	0.0%	0.0%	0.0% (0.0%, 0.0%)	u99IndSum01	27	0.0%	0.0%	0.0%	0.0% (0.0%, 0.0%)
u95Sum10	u95ProbSum10	16	6.4%	2.9%	9.1%	6.4% (2.9%, 9.1%)	u95IndSum10	19	19.9%	10.6%	27.6%	19.9% (10.6%, 27.6%)
u95Sum05	u95ProbSum05	17	0.8%	0.4%	2.1%	0.8% (0.4%, 2.1%)	u95IndSum05	20	5.5%	2.3%	11.5%	5.5% (2.3%, 11.5%)

## Results

**Figure 3:**
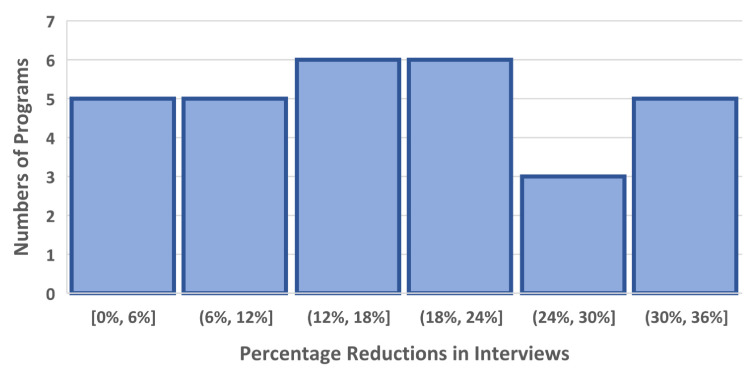
Numbers of programs with each listed percentage reductions in numbers of interviews Histogram of the percentage reductions in interviews achievable for each of the 30 studied programs. Interviews would not be given to applicants with a 99% upper prediction limit for a probability of matching that is less than 10%. The square bracket indicates "up to and including" and the round parenthesis indicates "up to but not including." For example, the second bar means values greater than 6% and less than or equal to 12%.

**Figure 4:**
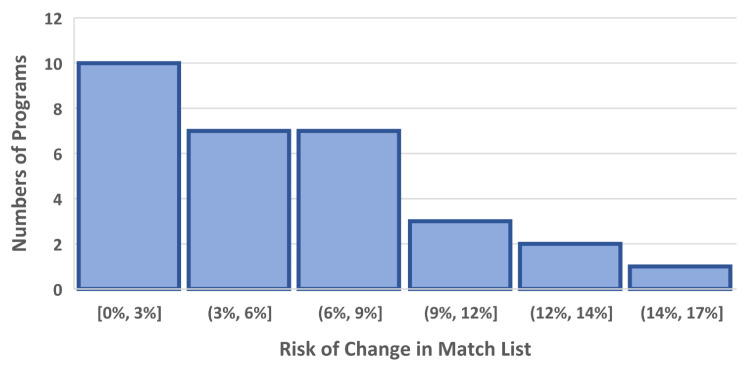
Histogram of risk induced by changing the match list Histogram of the risk of one or more changes in the match list from changing which applicants are given interviews. The change is that interviews would not be given to applicants with a 99% upper prediction limit for a probability of matching that is less than 10%. As explained in the Methods above equation (2), these listed probabilities are overestimates of the risk of one or more changes from removing these interviews. This figure shows substantive skewness among programs. For this reason, we calculate 97.5% two-sided confidence intervals for the median program (see Results) using a rank-based non-parametric method that is conservative (i.e., deliberately wide). Thus, there are two ways that we have deliberately assured that our results are conservative, both overestimating the probabilities and by our choice of confidence intervals for the median program. On the x-axis, the square bracket indicates "up to and including" and the round parenthesis indicates "up to but not including/".

In this four-year cohort of anesthesiology match cycles, the median residency program would benefit in its ability to reduce the number of interviews completed by approximately 16.9% (97.5% confidence interval 8.5%-24.1%; Table 1 and Figure 3). The table shows five reasonable criteria (column 1) that a program could choose to exclude marginal interviews. These numbers come directly from the data workbook provided as supplemental content. For example, suppose that all 30 programs had each been provided the upper 99% prediction limit for each applicant matching at the program. If the programs had then excluded applicants with prediction limits less than 10%, the median of the 30 programs would have had 16.9% fewer interviews (Figure 4). This would have come with an associated risk of a change in the match list of 5.3% (Figure 3). However, because there are N=30 programs, the median combines two numbers. In the corresponding worksheet, the program labeled 24 has both a 16.3% estimated reduction in interviews and a 5.4% risk of a change in the match list. The corresponding median savings would be 0.80 interviews per matched spot (0.34-1.33). These reductions are based on the threshold that the programs would not invite applicants with a 99% upper prediction limit for the probability of matching being less than 10.0%. In doing so, the median program would sustain a risk of approximately 5.3% of a change in the final match list (97.5% confidence interval 2.3%-7.9%; Figure 4). Programs with a greater potential benefit of reducing interviews would also have a greater risk of a change in their rank-to-match list; Spearman rank correlation 0.97 (95% confidence interval 0.93 to 0.99; Figure 3; StatXact 12.0, Cytel, Inc., Cambridge, MA).

Choosing a threshold of 1%, based on a 95% upper prediction limit, bears no utility since these cutoff criteria would identify no interviewees unlikely to match (Table 1). Similarly, reducing the individual interview probability threshold of not matching 5% yields the median program a reduction in interviews of only 4.9% (1.5%-8.9%).

## Discussion

**Figure 5:**
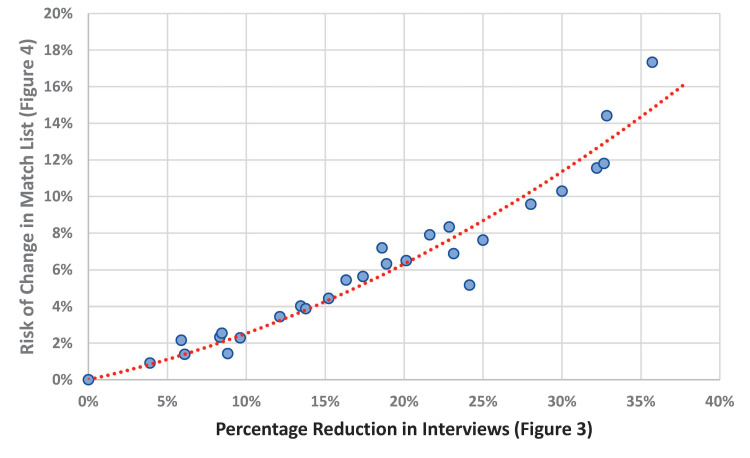
Risk of change in the match list by the increased percentage reduction in interviews The plot of the histogram from Figure 3 (horizontal axis) versus the histogram from Figure 4 (vertical axis). Individual programs with the greatest percentage potential gain by reducing interviews were also the programs with the greatest risk of change in the match list. Although intuitively reasonable, the strong monotonic relationship was unexpected.

Programs have increased the number of applicants interviewed, a trend which continued in the 2020-2021 recruitment season [18] possibly as a compensatory effort given the unique virtual recruitment season as a result of COVID-19. Our analysis strongly suggests that the median anesthesiology residency program was already over-interviewing by at least 8.5%, and more likely for approximately 1/6 of their interviewee list. The value of our work is the quantification of the specific extent to which interviews can reasonably be cut with small risk of changes in match lists (Figure 5).

Given the high costs of interviewing to both applicants and programs, reducing the total interviews for each residency program is a significant and specific recommendation for all stakeholders. This will drop per season costs for matched applicants, which typically exceeds $10,000 per individual [2,19-21], with the National Residency Matching Program (NRMP) reporting matched applicants ranking 10-11 programs [22]. Residency programs have reported spending approximately $150,000-200,000 per recruitment cycle, for the cost of the time of the program director and recruitment committee to review applications and perform interviews [9,23]. Using the midpoint, our findings could save programs approximately $30,000 ($15,000-$42,000). Thus, fewer total interviews would both result in a less costly match process for applicants and programs, achieved by eliminating the interviews very unlikely to result in a match.

Our prior work demonstrated the efficacy of modeling the probability of an interviewee matching with a program based on program and applicant characteristics [8]. This study extended that prior work by considering risk aggregated at the program level. Nominally, Table 1 enables program leadership to determine their own cut-offs for culling interviews based on risk tolerance or averseness. However, because the change from using a 99% upper prediction limit to 95% is based on the statistical model and not information available to the individual program, we would recommend consideration of changing the cutoff t. Naturally, when taking only the subset of interviewees who match, each additional interview that an interviewee completes results in a reduction in the probability of matching that single interview. As programs across the specialty interview more for a mostly finite supply of positions, the simple signals of application and interview give less and less assurance of matching. On the other hand, reducing the cutoff from 10% to 5% markedly reduces the potential benefit (Table 1).

In this study, we performed calculations by program. How the calculations would be used should not be literally an application of the results. For example, a program may have a candidate apply with an estimated low probability of matching but the value to the organization of their coming may be large (e.g., disadvantaged minority). As another example, an applicant may be overqualified for the program and have never even visited the state. However, the University has an investigator with which the applicant wants to do her postdoctoral fellowship. We find that the balance of risk and reward are best achieved through the highlighted row of Table 1. Figure 3 provides the insight that for the individual program there is a direct association between the extra interviewees and the risk of a change in the rank-to-match list.

The Coalition for Physician Accountability recently released recommendations to help guide the transition from undergraduate to graduate medical education that was admittedly lacking in an evidence base and formed primarily by consensus [24]. The objective of our work is to increase program and applicant co-awareness of match probability, with data-driven improvements and practices that should lead to a less costly, and less onerous application season. Two recently performed surveys of program directors did not solicit geographic location as an important factor for program directors in determining rank lists. Thus, we think that the survey study helps explain the reason for our finding of substantive potential savings in interviews - program directors currently are not taking the factor into account.

With an impending change in USMLE Step 1 reporting to Pass/Fail and increasing focus on diversity and equity, innovative metrics incorporating applicant-program fit are needed. This initiative requires that programs and applicants optimize interviews with the likelihood of match. We consider the "closeness" of interviewees to programs on dimensions of widely used measures of scholastic performance including board scores and membership to honor societies.

Limitations

The usefulness of our study is limited by the precision of the underlying probabilistic model (e.g., as shown by the differences in results based on 95% and 99% prediction limits). That model had a few drawbacks, chiefly that some of the data (about medical school ranking and AOA status) were frequently missing.

We do not directly consider race, ethnicity, or socioeconomic status. Therefore, we do not suggest that programs perform this analysis and then naively implement an interview threshold with no consideration of the nuances of candidates. Instead, we expect that these probabilities would be used as an aid to help programs feel more confident when determining who they should/not interview. If programs have particular recruiting criteria, such as attracting additional underrepresented minorities, programs can simultaneously consider our model's predictions as well as interviewees' demographics, and interview students who meet recruitment goals but are also relatively likely to match. Programs could also consider underrepresented minorities completely outside of this framework. The goal of this work is not to give programs a black box with which to blindly cull their interviewees but instead to provide an evidence-based tool to complement decision-making in recruitment. We then provide a summary of the potential reduction in interviews as a guide for programs to consider how to appropriately implement thresholds for their individual program.

Another notable limitation was that the model used a commonly reported field, “Current Address,” to determine whether an interviewee and interviewer were located in the same state. This, of course, misses some of the nuances of the geographic relationships between interviewees and programs. However, this model has been validated [8]. In the future, this work will be limited by the change in USMLE reporting. Beginning as early as 2022, USMLE Step 1 scores will move to pass/fail. In our prior study [8], the average of USMLE 1 and 2 CK was used as input. This was due to the high collinearity of these exams but not wanting to exclude data. We expect that these results will remain stable taking only USMLE Step 2CK scores into consideration.

## Conclusions

We aggregate the probabilities of select applicant and program characteristics in effecting a match to create a useful quantitative framework for programs to evaluate whether to invite applicants with a low probability of a match. We showed that a well-chosen decision rule nominally affects a program’s final match list when forgoing interviews with a low probability (less than 1%, 5%, or 10%) of matching. Our results of a median 16.9% reduction in interviews (0.80 interviews per matched spot) are novel because they are calculated using data on interviews and not only of match lists.
